# Efficient derivation and banking of clinical-grade human embryonic stem cell lines in accordance with Japanese regulations

**DOI:** 10.1016/j.reth.2022.10.006

**Published:** 2022-11-06

**Authors:** Kei Takada, Ryoko Nakatani, Emiko Moribe, Shizuka Yamazaki-Fujigaki, Mai Fujii, Masayo Furuta, Hirofumi Suemori, Eihachiro Kawase

**Affiliations:** Division of Clinical Basis for ES Cell Research, Center for Human ES Cell Research, Institute for Life and Medical Sciences, Kyoto University, 53 Kawahara-cho, Shogoin, Sakyo-ku, Kyoto, 606-8507, Japan

**Keywords:** Human embryonic stem cells, Clinical grade, Cell banking, hESCs, human embryonic stem cells, GMP, good manufacturing practice, QC, quality control, LM, laminin, CPF, cell processing facility, MHLW, Ministry of Health, Labour, and Welfare, ICM, inner cell mass

## Abstract

**Introduction:**

We recently established clinical-grade human embryonic stem cell (hESC) line KthES11 in accordance with current good manufacturing practice standards in Japan. Despite this success, the establishment efficiency was very low at 7.1% in the first period.

**Methods:**

To establish clinical-grade hESC lines, we used xeno-free chemically defined medium StemFit AK03N with the LM-E8 fragments instead of feeder cells. The protocol was then optimized, especially in the early culture phase.

**Results:**

We established five hESC lines (KthES12, KthES13, KthES14, KthES15, and KthES16) with 45.5% efficiency. All five hESC lines showed typical hESC-like morphology, a normal karyotype, pluripotent state, and differentiation potential for all three germ layers. Furthermore, we developed efficient procedures to prepare master cell stocks for clinical-grade hESC lines and an efficient strategy for quality control testing.

**Conclusions:**

Our master cell stocks of hESC lines may contribute to therapeutic applications using human pluripotent stem cells in Japan and other countries.

## Highlights

• We established five clinical-grade hESC lines without feeders at high efficiency.
• The hESC lines had typical hESC-like characteristics with a normal karyotype.
• We prepared master cell stocks of clinical-grade hESCs for therapeutic application.
• These clinical-grade hESC lines are ready to be distributed.

## 1 Introduction

Since the first hESCs were established in 1998 [1], their unique characteristics and potential for the development of regenerative medicine have fascinated many people. In Japan, we had established five hESC lines by 2008 [2,3] in a specifically designed facility, but not in keeping with current good manufacturing practice (GMP). The cell lines were established and maintained using feeder cells such as mouse embryonic fibroblasts and/or SL10 cells, a STO subline. Additionally, we used culture medium containing animal components. Thus, we aimed to establish new hESC lines under GMP-grade culture and operation systems for clinical use. Therefore, we built a cell processing facility (CPF) and prepared to establish clinical-grade hESCs, including the preparation of standard operating procedures.

Approximately 10 years after establishing hESC lines in 1998, Crook et al. in Singapore reported six clinical-grade hESC lines derived from embryos imported from Australia [4]. However, they still used animal products for the derivation. Since then, clinical-grade hESCs have been established in many countries, including the United States [5], United Kingdom [[6], [7], [8]], Israel [9], Finland [10], and China [11,12].

Clinical studies using human pluripotent stem cells (hPSCs) have been conducted in at least 10 countries, and significantly in the United States and China. By the end of 2019, there were at least 54 clinical studies of using hPSCs for the treatment of 22 diseases. Of these studies, 32 clinical studies are performed with hESC-derived cell products [13].

In Japan, “The Act on the Safety of Regenerative Medicine” and “Pharmaceuticals, Medical Devices, and Other Therapeutic Products Act” were enacted in November 2014 to realize and commercialize regenerative medicine [14]. Following these laws, it was possible to establish hESC lines for clinical use in Japan.

To establish hESC lines for clinical use, we chose E8 fragments of laminin (LM-E8s) instead of feeder cells. Rodin et al. and we showed that laminin-511/521 are a suitable alternative culture substrate to support hPSC culture compared with other extracellular matrix proteins and Matrigel [15,16]. Furthermore, we have demonstrated that LM-E8s provide superior adhesion for hPSC culture compared with intact laminin-511 and efficiently expand hPSCs with high quality and homogeneity [17].

We started to establish clinical-grade hESCs in 2017 and reported the first clinical-grade hESC line, KthES11, in 2018 [18]. During this period, the establishment efficiency was low at one-fourteenth. We had decided on items for quality control (QC) testing of hESCs for clinical use and were performing the tests, but when it was appropriate to perform QC testing was unclear.

In this study, we developed a system to establish clinical-grade hESC lines with high efficiency. Furthermore, we performed QC tests separately during the manufacturing process for master cell stocks.

## 2 Methods

### 2.1 Ethics statement

The study was approved by the ethics committee of Life and Medical Sciences, Kyoto University (approval number, ES-1), for derivation and characterization, including early differentiation of hESC lines from donated blastocysts, where informed consent was obtained from donors’ parents. Additionally, the study was approved by the Ministry of Education, Culture, Sports, Science, and Technology (license number: 0630) and the Ministry of Health, Labour, and Welfare (MHLW) (license number: 0630) in Japan.

### 2.2 Facility summary

Derivation of clinical-grade hESC lines and their banking were performed in our CPF operated with GMP-level management. In line with “The Act on the Safety of Regenerative Medicine,” the facility obtained a manufacturing license (#FA5160004) from MLHW after passing a site visit by the Pharmaceuticals Medical Devices Agency in Japan. Long-term culture and QC tests of our master cell stocks were performed outside the CPF.

### 2.3 Isolation and culture of clinical-grade hESCs

We gently isolated the inner cell mass (ICM) from day 5–6 blastocysts using a micromanipulator (Narishige, Tokyo, Japan). The ICMs were seeded on a Falcon Center Well Organ Culture Dish (Corning, NY, USA) precoated with LM-E8s (0.5 μg/cm^2^, iMatrix-511MG, Matrixome, Osaka, Japan) in StemFit AK03N (Ajinomoto, Tokyo, Japan) with Y-27632 (final concentration: 10 μM; FUJIFILM Wako, Osaka, Japan). Although ICMs had fully attached by the next day, we used Y-27632 during ICM culture for the initial several passages. At approximately 7 days, ICMs with outgrowths were mechanically dissected into small pieces and transferred into a new culture well precoated with iMatrix-511MG. After 14–16 days of culture in an incubator at 37 °C with 5% CO_2_ and 5% O_2_, morphologically hESC-like cells had appeared. The cells were subsequently cultured for three to four passages by mechanical dissection using 31 G needles, and the resulting cells appeared to be stabilized as hESCs. At this stage, the hESCs were capable of passaging as small clumps using a non-enzymatic solution (5 mM EDTA in PBS) and cryopreservation for early stocks. When seeded at 1–2 × 10^5^ cells/well (6-well plate), the cells became confluent every 3–4 days. During culture, cell viability was examined at each passage by a Via1-Cassette™ with NucleoCounter NC-200 (ChemoMetec, Allerød, Denmark). Cell viability was generally higher than 75%.

### 2.4 Cell banking strategy

hESC lines at early passages were harvested in a batch of five to 10 vials as seed stocks with 5 × 10^5^ to 2 × 10^6^ viable cells per vial. To prepare master cell stocks, we thawed one vial from seed stocks, expanded the cells in culture, and then banked the cells in batches of 50 vials with >1 × 10^6^ viable cells per vial in cryopreservation medium consisting of StemFit AK03N with 10% DMSO (Sigma–Aldrich, St. Louis, MO, USA) and Y-27632 (final concentration; 5 μM). The number of viable collected cells was determined by double staining with acridine orange and DAPI (ChemoMetec). The vials were labeled for traceability and placed in a CoolCell freezing container (Biocision, San Rafael, CA, USA) in a −80 °C mechanical freezer (PHC, Tokyo, Japan). The next day, the vials were transferred to the vapor phase of a liquid nitrogen tank (LN2 supply tank, Taiyo Nippon Sanso, Tokyo, Japan).

### 2.5 Quality control testing of clinical-grade hESC lines

Current drafts for QC testing are described in Table 1. Our QC testing was based on the guidelines of the International Stem Cell Banking Initiative [19]. We used four categories: (1) in-process, (2) cell authenticity, (3) biological safety, and (4) characterization and potency. For example, we performed photomicrography daily to confirm that >80% of colonies were undifferentiated. When hESCs were subcultured, we examined the total cell number and viability. In general, cell viability was >75%. As cell authenticity tests, HLA and STR tests were performed during master cell stock preparation.

**Table 1:** Summary of QC testing for clinical-grade hPSCs.

Examination items	Analytical method	Equipment and reagents	Acceptance criteria	QC tests		
**1) In-process tests**				**1**	**2**	**3**
Morphology	Visual confirmation		Undifferentiated: >80% of colonies	○	○	○
Cell count	Cell count	NucleoCounter (ChemoMetec) NC-200/Via-1 Cassette (ChemoMetec)		○	○	○
Viability	Acridine Orange/DAPI staining	NucleoCounter (ChemoMetec) NC-200/Via-1 Cassette (ChemoMetec)	>75%	○	○	○
**2) Cell authenticity tests**						
HLA	Sequencing-based typing (SBT) (outsourced)		Identical		○	
STR	STR (including outsourcing)	GenomeLab GeXP/Human STR kit (SCIEX)	Identical		○	
**3) Biological safety tests**						
Sterility	In accordance with the current requirements of the Japanese Pharmacopoeia	Tryptic Soy SCD (Anaerobiotic, Aerobiotic)	Negative	○	○	(○)
Mycoplasma	Culture methods, qualified qPCR	PrepSEQ and MycoSEC (Thermo Fisher Scientific)	Negative		○	
Endotoxin	Kinetic-colorimetric method Kinetic turbidimetric method in accordance with the Japanese Pharmacopoeia (outsourced)	Endosafe PTS (JP) (Charles River)	Negative		○	
Karyotype	G-banding	Ikaros, Isis (Zeiss)	Normal (diploid) ≥20 metaphases	○	○	○
Viral testing	PCR (outsourced)		HBV, HCV, HIV, HTLV, EB, HSV, HHV-6, HHV-7, CMV, HPV, and Parvo-B19: all negative		○	
**4) Characterization and potency tests**						
Marker expression of hPSCs	Immunocytochemisty	Oct3/4, Nanog, and TRA-1-60	Positive (>80% of cells)	(○)	(○)	○
Marker expression of hPSCs	Flow cytometry	BD Human pluripotent stem cell analysis kits (#560461, 560477, and 560589) with TRA-1-60 antibody (560193)	SSEA-1: negative (≤10%) Other markers: positive (≥70%)	(○)	(○)	○
Differentiation potential	Embryoid body formation and scorecard	TaqMan hPSC Scorecard Panel (Thermo Fisher Scientific)	Positive for all three germ layers	(○)	(○)	○

For biological safety tests, sterility tests were performed during both seed stock and master cell stock preparations. Other biological safety tests were performed during master cell stock preparation.

Conversely, characterization and potency were examined as long-term culture tests from more than three independent trials using our master cell stocks. Karyotype analysis was performed using seed and master cell stocks, and after every five passages during long-term culture tests. Detailed QC testing protocols have been described previously [8].

For ectodermal differentiation, we used STEMdiff™ Trilineage Ectoderm Medium (STEMCELL Technologies, Vancouver, Canada) in accordance with the manufacturer's instructions except that the number of induction days was extended from 7 to 11 days.

For neuronal cell differentiation, we used a Quick-Neuron™ Dopaminergic - mRNA Kit (Elixirgen Scientific, Baltimore, MD USA) in accordance with the manufacturer's instructions. Then, the cells were fixed in 4% paraformaldehyde, permeabilized with 0.2% Triton X-100 in PBS, and stained with the primary and secondary antibodies described in Supplementary Table 1. We performed antibody incubations for 1 h at room temperature or overnight at 4 °C. Nuclei were counterstained with DAPI (Thermo Fisher Scientific).

ABO genotyping was performed by allele-specific PCR as described previously [20] with the following modifications. Genomic DNA from each cell line was purified using a FlexiGene DNA Kit (Qiagen) in accordance with the manufacturer's instructions. Real-time PCR using 1 ng DNA was carried out with Power SYBR Green Master Mix (Thermo Fisher Scientific) and the StepOnePlus™ Real-Time PCR System (Thermo Fisher Scientific). Thermal cycling was denaturation at 95 °C for 10 min, followed by 40 cycles of 95 °C for 10 s, 60 °C for 20 s, and 72 °C for 30 s.

## 3 Results

### 3.1 Workflow of derivation, characterization, and banking of clinical-grade hESCs

To prepare stocks efficiently in a compact space with a small number of people, we developed a system for clinical-grade hESC banking as shown in Fig. 1, which was characterized by the following points.

1. Six to 10 weeks were required to produce master cell stocks from 5–6-day blastocysts. Therefore, we prepared seed stocks before the master stock and divided the manufacturing process into two parts to allow for flexibility in the manufacturing schedule. We generally prepared five to 10 frozen vials as seed stocks.
2. We performed QC tests separately in stages as shown in Table 1 and Fig. 1. Cell authenticity and biological safety were examined during master cell stock preparation in the CPF. Conversely, other characterizations, such as hESC marker expression and differentiation potential, were performed using long-term cultured cells from master cell stocks with thawing (QC test 3).
3. Long-term culture tests were performed in a specially designated facility with an air conditioning system with a HEPA filter. All materials and solutions were the same grades as those in the CPF.
4. Before master cell stock preparation, we continuously cultured some hESCs from seed stock preparation outside the CPF and developed optimal culture protocols for each cell line. Furthermore, we performed some QC tests, such as gene expression analysis and flow cytometry, to confirm that seed stocks had some hESC characteristics in addition to their morphology.
5. Karyotype analyses were carried out during master cell stock preparation and every five passages during long-term culture.

**Fig. 1:**
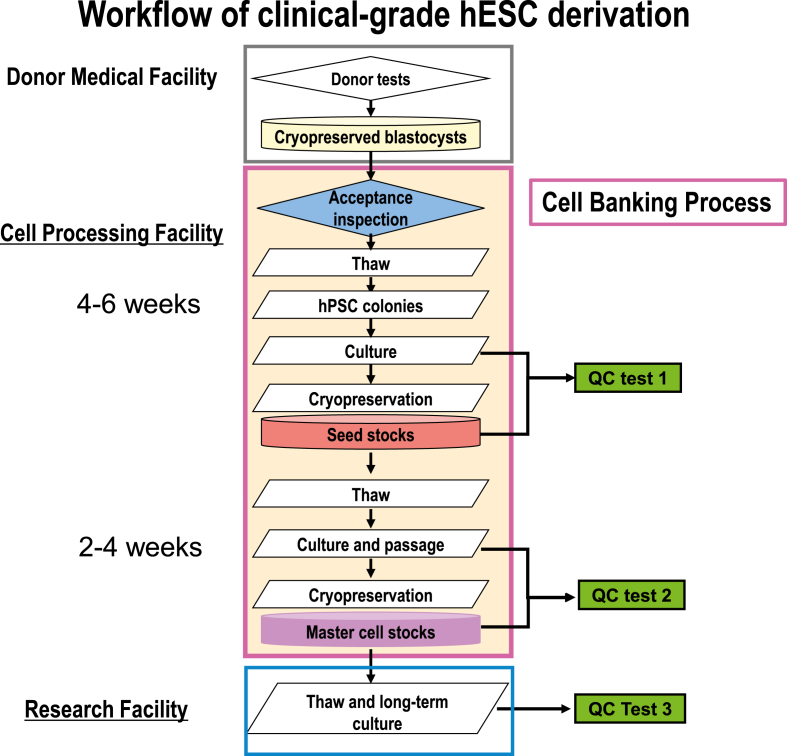
Workflow of clinical-grade hESC derivation. Our cell processing facility used intermediated seed stocks to derive hESCs from a blastocyst, and then prepared master cell stocks. We separated quality control (QC) tests 1, 2, and 3, where the items are listed in Table 1.

### 3.2 Establishment of clinical-grade hESCs without feeder cells

We established only one clinical-grade hESC line, KthES11, from 14 blastocysts in period 1. One major problem was poor adhesion of the ICM to LM-E8s. We previously showed that LM-E8s promote strong adhesion of hPSCs compared with other culture substrates, such as Matrigel and intact laminin [17], suggesting that the remaining trophectoderm cells were not suitable for adhesion to LM-E8s. Therefore, we carefully removed the trophectoderm area from blastocysts using micromanipulators. As expected, proper ICMs adhered well to LM-E8s until the next day.

Another major issue was the sensitivity against subculturing hESC-like cells during initial passages, even when we used non-enzymatic 5 mM EDTA/PBS and added Y-27632 to the culture medium. Therefore, we applied mechanical dissociation using 31 G needles for the stages. After hESC-like cells had appeared to stabilize with a sufficient number (passage 3 to 4), the cells were passaged as small colonies using 5 mM EDTA in PBS as described in the Materials and Methods.

With these improvements, our establishment efficiency was increased to 45.5% (five out of 11) in Period 2 from 7.1% (one out of 14) in Period 1 (Table 2).

**Table 2:** List of clinical-grade hESC lines established at the Institute for Life and Medial Sciences, Kyoto University.

Period	No. of blastocysts used	No. of hESC lines established	Efficiency of establishing hESC lines (%)	hESC line	Unique cell line identifier	Karyotype	Blood type	Culture Condition	Date of official establishment
I	14	1	7.1	KthES11	KUIMSe004-A	46, XX	AB	StemFit AK03N/iMatrix511MG	May 7, 2018
II	11	5	45.5	KthES12	KUIMSe005-A	46, XX	AO	StemFit AK03N/iMatrix511MG	December 7, 2018
				KthES13	KUIMSe006-A	46, XX	AO	StemFit AK03N/iMatrix511MG	April 11, 2019
				KthES14	KUIMSe007-A	46, XX	AO	StemFit AK03N/iMatrix511MG	December 5, 2019
				KthES15	KUIMSe008-A	46, XY	O	StemFit AK03N/iMatrix511MG	June 25, 2020
				KthES16	KUIMSe009-A	46, XX	AO	StemFit AK03N/iMatrix511MG	April 12, 2021

Furthermore, we established clinical-grade hESCs using iMatrix-511MG at a concentration of 0.5 μg/cm^2^, but the cells could be generally maintained using iMatrix-511MG at a lower concentration (e.g., 0.2–0.25 μg/cm^2^). At this concentration, cell viability during subculture was approximately 90%.

### 3.3 Characterization of clinical-grade hESC lines

**Fig. 2:**
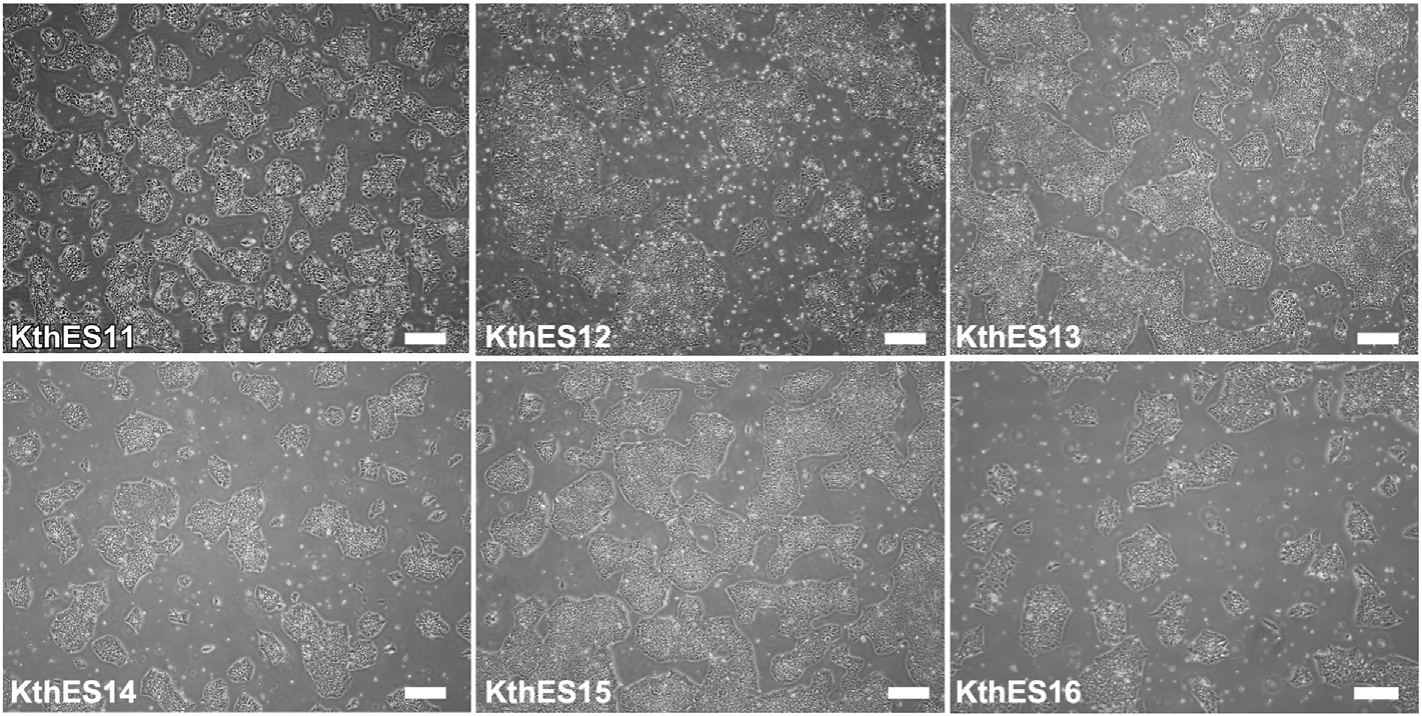
Phase contrast microcopy of clinical-grade KthES cells. All KthES cell lines showed typical hESC morphology. Scale bar, 300 μm.

**Fig. 3:**
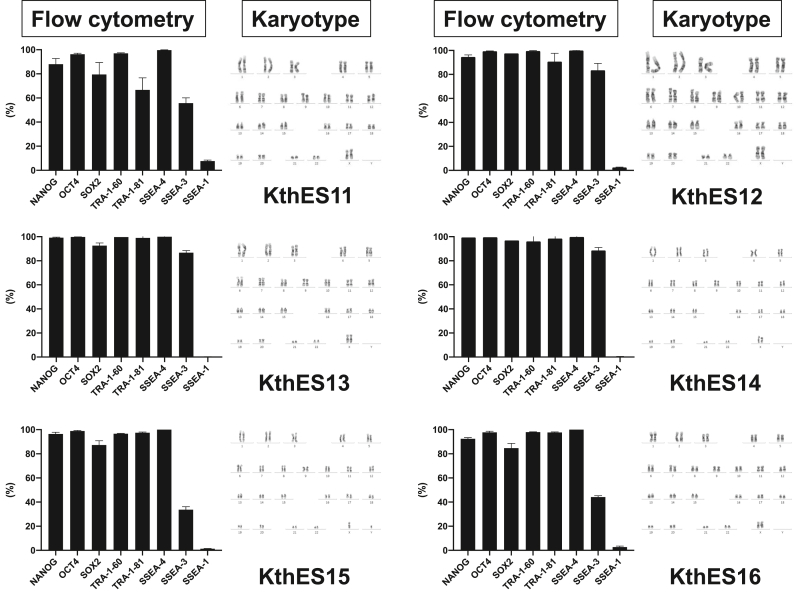
Qualification of KthES cell lines after long-term culture using our master cell stocks. (Left) Flow cytometric analysis indicated high expression of pluripotent cell markers: transcriptional factors (NANOG, OCT4, and SOX2) and cell surface markers (TRA-1-60, and TRA-1-81 and SSEA-4). SSEA-3 expression varied from moderate to high among the cell lines. The differentiation marker SSEA-1 showed low expression. The vertical axis is the percentage of positive cells among total cells (%). (Right) Representative G-banding karyotype analysis of the six KthES cell lines. All KthES cell lines had a normal karyotype. KthES15: XY, other cell lines: XX.

In the second period, we established another five clinical-grade hESC lines of both sexes with different blood types (Table 2). We had already reported the KthES11 line [18], but had not evaluated the cells in long-term culture. Therefore, we evaluated the cells in conjunction with the five hESC lines established in this study. All hESC lines showed typical hESC-like morphology (Fig. 2). Flow cytometric analysis also revealed high expression of pluripotent cell markers of transcriptional factors (NANOG, OCT4, and SOX2) and cell surface markers (TRA-1-60, TRA-1-81, and SSEA-4) (Fig. 3). Cell surface marker SSEA-3 showed moderate to high expression depending on the cell line. Conversely, the differentiation marker SSEA-1 showed low expression.

For karyotype analysis, we performed G-banding every five passages from our seed stocks. All six cell lines showed normal karyotypes in long-term culture (Fig. 3).

**Fig. 4:**
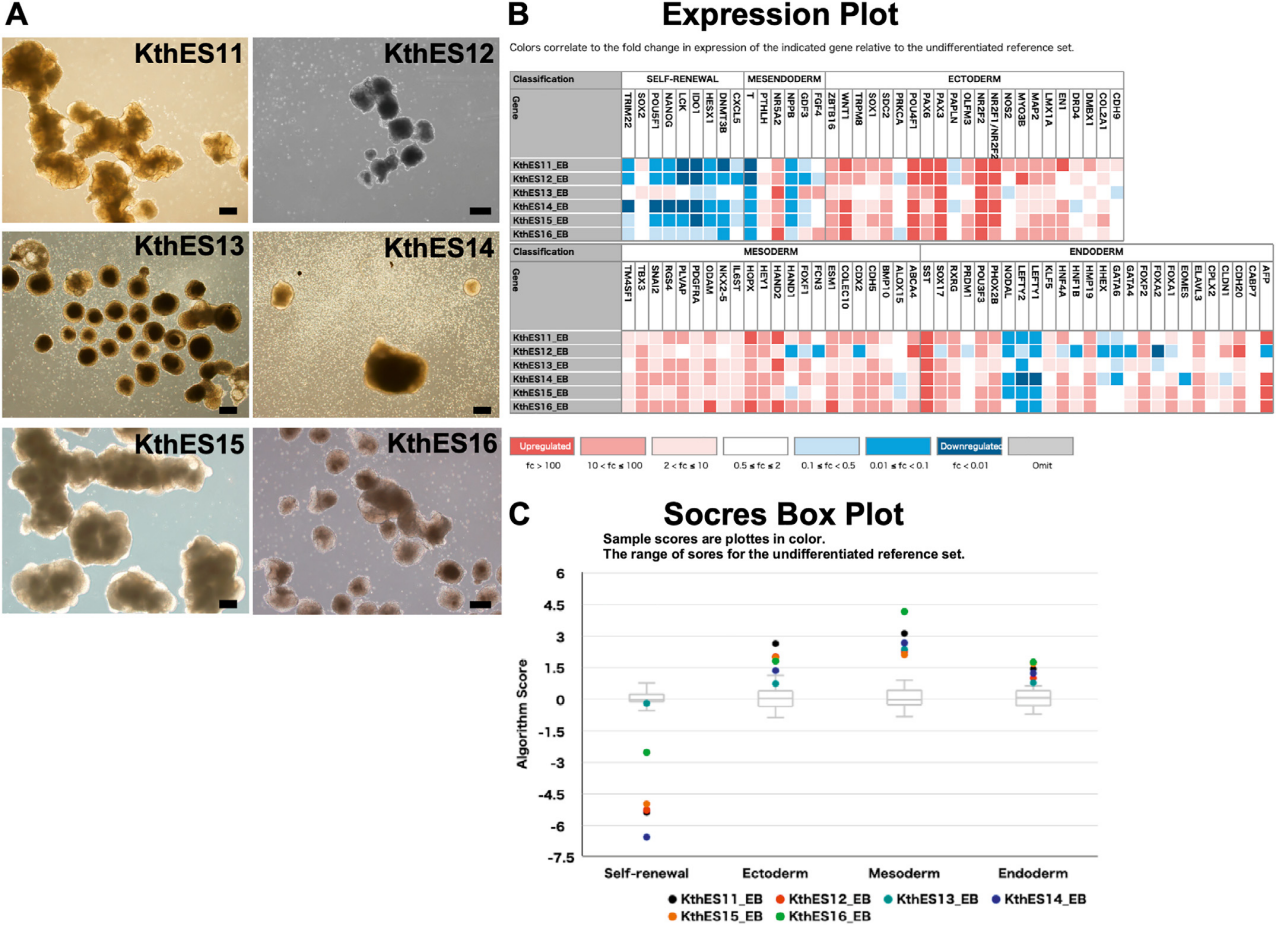
Qualification of the differentiation potency of KthES cell lines after long-term culture using master cell stocks. All KthES cell lines formed EBs using an Aggrewell™ 800 (STEMCELL Technologies) and culture in custom mTeSR™1 (without bFGF and TGFb, STEMCELL Technologies) with Ultra-low attachment plates (Corning). The cell lines showed a differentiation potential evaluated by the TaqMan hPSC Scorecard Panel (Thermo Fisher Scientific). (A) Embryoid body formation in KthES cell cultures differentiated for 14 days. Scale bar, 300 μm. (B, C) Results of the TaqMan hPSC Scorecard Panel. (B) Expression plot and (C) score box plot.

To assess pluripotency, hESCs in long-term culture were grown in suspension in custom mTeSR™1 (without bFGF and TGFb; STEMCELL Technologies). The cells formed embryoid bodies (EBs) (Fig. 4A), and then the EBs expressed markers of all three germ layers as confirmed by the TaqMan hPSC Scorecard Panel (Thermo Fisher Scientific) (Fig. 4B). The score box plot shows that the expression of markers for all three germ layers derived from the six clinical-grade hESC lines was increased significantly, except for KthES13 cells that had elevated expression of ectoderm markers, but not at a significant level (Fig. 4C). Therefore, we examined ectoderm differentiation in KthES13 cells using STEMdiff™ Trilineage Ectodermal Medium (STEMCELL Technologies). In this system, the score of ectoderm markers became significant (Supplementary Figs. 1A and B). Furthermore, KthES13 cells differentiated into dopaminergic neurons using a differentiation kit (Elixirgen Scientific) (Supplementary Fig. 1C). Taken together, we concluded that KthES cells had a differentiation potential for all three germ layers.

Our master cell stocks of all clinical-grade hESCs were sterile, free of mycoplasma, and negative for the viruses described in the Materials and Methods. Furthermore, the cell lines passed an endotoxin test. These data are available from the corresponding author.

## 4 Discussion

We established five clinical-grade hESC lines without feeders at high efficiency. Furthermore, we developed an efficient system for QC testing. All five hESC lines were pluripotent with normal karyotypes. The hESC lines also showed a differentiation potential in an EB formation assay and other assessment methods.

ICM is inside the blastocyst and covered with trophectoderm, which expresses integrin αVβ3 [21,22]. Accordingly, fibronectin or vitronectin is suitable to adhere the trophectoderm, but not laminin. Therefore, we isolated ICMs by gentle removal of the trophectoderm, and then most ICMs attached well.

We used a non-enzymatic EDTA solution for subculture. This method does not dissociate cells into single cells and appears to be less damaging than dissociation by enzymatic solutions such as TrypLE Select (Thermo Fisher Scientific). Although there was good cell viability with normal passaging, cell viability was often low in the first phase of the establishment process. The preparation of master cell stock was often very slow. Therefore, we dissociated the cells mechanically when cultured in a center well organ culture dish, i.e., when the number of cells was small. These changes greatly improved the efficiency of establishment.

Our aims when establishing clinical-grade hESC lines consisted of three evaluation stages: preparation of seed stocks, preparation of master cell stocks, and evaluation by long-term culture using the master cell stocks. We did not perform all QC tests for every step, but they were assigned to each stage. When we prepared seed stocks, we performed sterility tests for biological safety. We also cultured the cells outside of the CPF using surplus cells from cryopreservation. We confirmed that the cell lines had a normal karyotype by G-binding. We examined whether the cell stocks had hESC characteristics by FACS analysis and immunocytochemistry. The reason why the established cells were hESCs was because of their morphology. Additionally, we optimized the culture method. For some cell lines, the concentration of iMatrix-511MG was lowered to 0.25 μg/cm^2^ to facilitate cell detachment and increase cell viability in passages. These pretests led to efficient preparation of the master cell stocks and QC testing.

Next, when preparing the master cell stocks, biological safety tests were the focus. In period 1, we used antibiotics in the medium during early culture. Therefore, we examined antibiotic residues using the highly sensitive LC/MS/MS method. Subsequently, we did not add antibiotics and did not need to conduct such a test. Evaluation tests for long-term culture were conducted outside of the CPF in a specially designated facility with an air conditioning system that used HEPA filters, while the reagents used for culture were the same as those used in the CPF. We have frozen and stored some of the master cell stocks and long-term cultured cells as evaluation samples for further development of QC testing.

Currently, we are distributing these clinical-grade hESC lines upon request. We expect to reveal more characteristics of our hESC lines through collaboration with our distribution partners. For example, we may find unique characteristics of the cell lines that can easily differentiate into specific cell types, cell lines that maintain genome stability even after long-term culture, or cell lines suitable for genetic recombination.

Because of the great potential of hESCs, many hESC lines have been established worldwide and have been registered in hPSCreg (https://hpscreg.eu/search?cell-type=hesc), in which we reported our cell lines, including those in this study. However, in Japan, only two centers, our institute and the National Research Institute for Child Health and Development [23], have succeeded in establishing hESC lines. Therefore, we have been entrusted with an important task: to establish hESC lines in Japan.

## 5 Conclusions

We established hESCs for clinical use with high efficiency. Our master cell stocks of the hESC lines may contribute to therapeutic applications using hESCs in Japan and other countries.

## Declaration of competing interest

The authors declare no competing interests.

## Figures and Tables

**Figs1:**
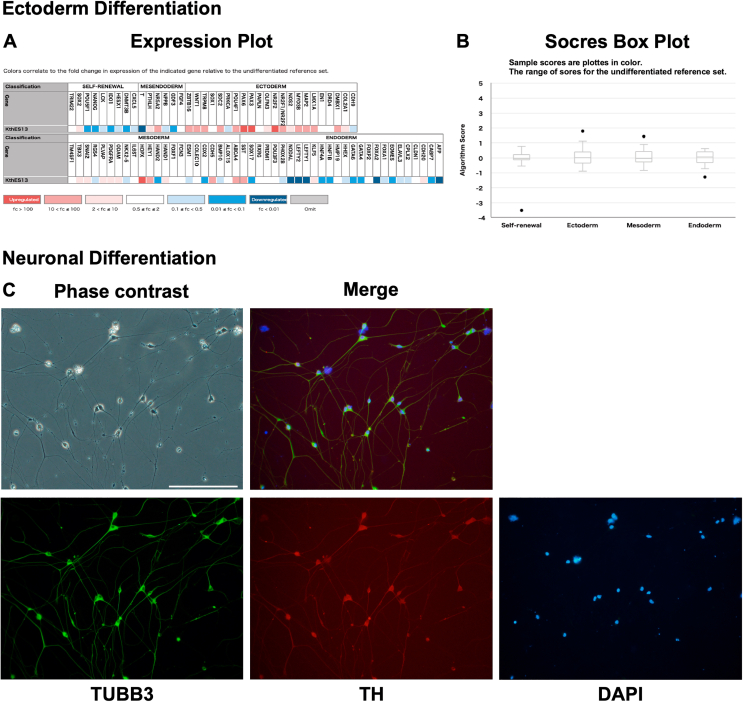
Supplementary Fig. 1. Ectodermal (A, B) and neuronal (C) differentiation potential of KthES13 cells. (A, B) KthES13 cells were cultured in STEMdiff™ Trilineage Ectoderm Medium and then differentiation potential was examined by the TaqMan hPSC Scorecard. Under this culture condition, the cells showed expression of ectoderm and mesoderm markers. (A) Expression plot and (B) Score box plot. (C) Immunofluorescence of KthES13 cells differentiated into dopaminergic neurons by Elixirgen Scientific technology. The cells showed typical neurite growth (phase contrast image) and expression of the pan-neuronal marker β-III tubulin (TUBB3) (green) on day 7 post-differentiation. Dopaminergic neurons were identified by an anti-tyrosine hydroxylase (TH) antibody (red). Nuclei were counterstained with DAPI (blue). The merged image is a superimposition of the three fluorescence images. Scale bar, 200 μm.
