# Supplementary material for: Efficient derivation and banking of clinical-grade human embryonic stem cell lines in accordance with Japanese regulations
Source: Regen Ther. 2022 Nov 6;21:553–9. doi: 10.1016/j.reth.2022.10.006 (PMC9647332; doi:10.1016/j.reth.2022.10.006)
Supplement: Multimedia component 1 [file mmc1.docx]

**Supplementary Table 1: Antibodies used for immunocytochemistry**

| **Antibody** | **Dilution** | **Company Cat #** | **RRID** |
| --- | --- | --- | --- |
| Mouse Anti-β-III tubulin monoclonal antibody | 1:250 | R and D Systems Cat# MAB1195 | AB_357520 |
| Rabbit Anti-TH primary antibody | 1:1000 | Abcam Cat#ab6211 | AB_2240393 |
| Alexa Fluor 488 goat-anti Mouse IgG (H+L) | 1:500 | Thermo Fisher Scientific Cat# A-11029 | AB_2534088 |
| Alexa Fluor 568 goat-anti Rabbit IgG (H+L) | 1:500 | Thermo Fisher Scientific Cat# A-11036 | AB_10563566 |
